# Development of the SIMPLE scoring system for predicting malignant brain edema in patients with large hemispheric infarction following acute ischemic stroke

**DOI:** 10.1007/s10143-025-04021-y

**Published:** 2026-01-06

**Authors:** Songrit Vuttipongkul, Ekawut Chankaew, Bunpot Sitthinamsuwan

**Affiliations:** Division of Neurosurgery, Department of Surgery, Faculty of Medicine Siriraj Hospital, Mahidol University, 2 Wang Lang Road, Bangkok Noi, Bangkok, 10700 Thailand

**Keywords:** Computed tomography, Infarct volume, Large hemispheric infarction, Malignant brain edema, Predictive score

## Abstract

Malignant brain edema (MBE) is a life-threatening complication of large hemispheric infarction (LHI). While early decompressive hemicraniectomy has been shown to improve outcomes in selected patients, identifying those at risk for MBE remains a clinical challenge. This study aimed to develop a simple and practical scoring system based on non-contrast CT imaging obtained approximately 24 h after stroke onset to support early prediction and timely intervention. We conducted a retrospective cohort study of LHI patients who underwent non-contrast CT imaging within 24 ± 8 h of symptom onset. Radiographic parameters assessed included infarct volume (ABC/2 method), ASPECTS, anterior or posterior cerebral artery territory involvement, temporal lobe involvement, midline shift, lateral ventricle compression, intercaudate distance, Sylvian fissure effacement and sulcal effacement. Multivariate logistic regression identified independent predictors of MBE. A weighted scoring system was then developed based on the regression model and internally validated using ROC analysis. Among 62 patients included, 38 (61.3%) developed MBE. Infarct volume and midline shift were independently associated with MBE. A scoring system (SIriraj Malignant Brain Edema Prediction by Lesion Volume and Edema Shift – SIMPLE) was derived using infarct volume ≥ 106 mL (two points) and midline shift ≥ 1.6 mm (one point). The score demonstrated excellent discriminative performance (AUC 0.951, 95% CI: 0.899–1.002). A cutoff score ≥ 2 yielded 89.5% sensitivity and 91.7% specificity. A dual-threshold strategy, using score 0 to rule out and score three to rule in, achieved 97.4% sensitivity and 100% specificity, providing clear clinical guidance for observation, medical management, or early neurosurgical intervention. The SIMPLE score is a rapid and reliable tool using routine CT imaging to predict MBE in LHI patients at an optimal 24-hour timepoint. Its simplicity and strong predictive accuracy may assist clinicians in early risk stratification and timely treatment decisions. Further prospective validation is recommended.

## Introduction

Ischemic stroke is one of the most prevalent neurological disorders and remains a major cause of disability and mortality worldwide. Large hemispheric infarction (LHI) accounts for approximately 10% of ischemic strokes and carries a high risk of progressing to malignant brain edema (MBE), which can result in increased intracranial pressure that leads to severe neurological deterioration, or death. Research has shown that approximately 80% of LHI patients will develop MBE [1, 2].

The current standard treatment for severe brain swelling in ischemic stroke patients is decompressive hemicraniectomy, a surgical intervention aimed at reducing intracranial pressure and saving lives. Multiple studies support the use of early decompressive hemicraniectomy (within 48 h of stroke onset), especially in patients younger than 60, as a means of improving survival and functional outcomes [3–7]. However, early decompressive hemicraniectomy has not been universally adopted as standard clinical practice, in part because not all LHI patients develop MBE. Applying a uniform protocol to operate within the early window could therefore result in some patients undergoing unnecessary surgery.

Moreover, a review of multiple studies suggests that the critical factor influencing better patient outcomes may not be the timing of decompressive hemicraniectomy in absolute hours, but rather whether the surgery is performed before the onset of brain herniation [8–12]. This is consistent with current theoretical understanding that MBE following ischemic stroke arises from cytotoxic edema within infarcted brain tissue, leading to increased intracranial pressure, brain herniation, and subsequent secondary brain injury [13]– [14]. Performing decompressive hemicraniectomy after herniation — referred to as late decompressive hemicraniectomy, which remains common in clinical practice — may be less effective due to factors such as delays in symptom recognition and surgical team readiness. These delays can prolong the herniation period and increase the risk of secondary brain injury.

To address this challenge, several predictive models have been proposed to estimate the risk of MBE in LHI patients [15–20]. However, a widely accepted, standardized scoring system has yet to be established [21]. This may be attributed to factors such as reliance on advanced imaging modalities like CT/MR perfusion, limited predictive accuracy, the fact that some models do not directly predict brain herniation, which is an essential factor in surgical decision-making, and discussion with patients’ families.

Therefore, the aim of this study is to develop a simple and practical scoring system based solely on radiographic evaluation using computed tomography (CT) imaging at a specific time point to predict the likelihood of developing MBE. To achieve this, our approach emphasizes CT scan parameters that are routinely available and widely accessible in clinical settings. These include factors previously shown to be statistically significant, such as infarct volume, involvement of Alberta Stroke Program Early CT Score (ASPECTS) regions, anterior cerebral artery (ACA) or posterior cerebral artery (PCA) territory involvement, temporal lobe involvement, sulci effacement, Sylvian fissure effacement, lateral ventricle compression, and midline shift [22–27]. Moreover, we investigated a potentially relevant but less frequently studied factor in LHI patients: the intercaudate distance. This parameter may reflect intracranial volume reserve and has been previously linked to MBE [25, 28]. By providing a more accessible and clinically feasible tool, this predictive model may assist clinicians in timely treatment decision-making, improve patient outcomes, and help reduce unnecessary surgical procedures.

## Materials and methods

### Study design and ethical approval

This was a single-center, retrospective observational study conducted at Siriraj Hospital, Mahidol University. The study included patients diagnosed with LHI who were referred for neurosurgical consultation. Data were collected through retrospective chart review from January 2008 to May 2023. Ethical approval was obtained from the Siriraj Institutional Review Board (SIRB), Faculty of Medicine Siriraj Hospital, Mahidol University, Bangkok, Thailand (Certificate of Approval (COA) number Si 516/2023). All patient data were kept confidential in accordance with the Declaration of Helsinki.

### Patient selection

This study included patients aged 18 years or older diagnosed with acute ischemic stroke confirmed by either CT or MRI, demonstrating infarction involving the middle cerebral artery (MCA) territory. The exclusion criteria were as follows: small cerebral infarction, presence of intraparenchymal hemorrhage affecting 30% or more of the infarcted territory with associated mass effect, coexisting brain tumors or other intracranial mass lesions, early malignant brain edema, patients who underwent early decompressive hemicraniectomy, defined as surgery performed prior to clinical or radiographic evidence of brain herniation, those who did not undergo a CT brain scan within 16 to 32 h (24 ± 8 h) after symptom onset, those who died before 24 h after stroke onset, and those with incomplete outcome data.

### Data collection

The following variables were collected and analyzed:

1. **Demographic and clinical data** including age, sex, vascular risk factors such as hypertension, diabetes mellitus, atrial fibrillation, and smoking history, time from stroke onset (in hours), systolic and diastolic blood pressure (SBP, DBP), body temperature, serum glucose level, National Institutes of Health Stroke Scale (NIHSS) score, Glasgow Coma Scale (GCS) score at admission, side of infarction (left or right) and stroke subtype (cardioembolic, large vessel occlusion, or undetermined).
2. **Radiographic variables** measured in this study included infarct volume (calculated using the ABC/2 method based on maximum width, perpendicular length, and height of the lesion on axial CT images) [29], ASPECTS [16,30], involvement of the anterior cerebral artery (ACA) or posterior cerebral artery (PCA) territories, temporal lobe involvement, midline shift (in mm), lateral ventricle (LV) compression, sulci effacement, Sylvian fissure effacement, and intercaudate distance.
3. **Treatment data** containing information about history of intravenous thrombolysis, mechanical thrombectomy, intravenous mannitol administration, and performance of decompressive hemicraniectomy.
4. **Clinical outcomes** including Glasglow Coma Scale (GCS) score and modified Rankin Scale (mRS) score at discharge or at 30 days post-onset. While functional outcomes were not the primary endpoint, they were documented for descriptive analysis.

Radiographic variables were evaluated using CT brain imaging performed between 16 and 32 h (24 ± 8 h) after stroke onset. In cases where multiple CT scans were available within this time window, the scan closest to 24 h was used for analysis.

The primary outcome was the development of MBE. The diagnosis of MBE was based on clinical and/or radiographic criteria. Clinical criteria included a decline in consciousness, defined as a drop in Glasgow Coma Scale (GCS) score by ≥ 2 points without an alternative identifiable cause, and/or the presence of anisocoria. Radiographic criteria included a midline shift ≥ 5 mm at the level of the septum pellucidum on CT imaging and/or evidence of uncal herniation.

## Reliability of radiographic variable measurement

The radiographic variables required for measurement included infarct volume (calculated by the ABC/2 method), midline shift, and intercaudate distance. These parameters were measured by two independent authors (S.V. as rater 1 and B.S. as rater 2). Intraclass correlation coefficient (ICC) for assessment of inter-rater reliability of an individual parameter is demonstrated as follows:

- Infarct volume (measured by the ABC/2 method): ICC 0.943, *p* < 0.001 (excellent agreement).
- Midline shift (direct measurement): ICC 0.972, *p* < 0.001 (excellent agreement).
- Intercaudate distance (direct measurement): ICC 0.876, *p* < 0.001 (good agreement).

## Treatment of ischemic stroke

The standard initial treatment for patients with acute ischemic stroke included intravenous thrombolysis or mechanical thrombectomy, when indicated. Follow-up non-contrast CT brain imaging was routinely performed after initial treatment to assess the extent of brain infarction. Patients found to have large territory infarctions were referred to the neurosurgical team for further evaluation.

The neurosurgery team assessed each patient to determine appropriate management strategies. Most patients were closely monitored for signs of MBE or impending brain herniation, based on clinical deterioration and radiographic changes. During the observation period, some patients received intravenous mannitol to reduce intracranial pressure as part of medical management. If clinical signs of brain herniation were identified, such as decreased level of consciousness, anisocoria, or increased midline shift, decompressive hemicraniectomy was considered as a treatment option. In some cases, the patient’s family declined surgical intervention and opted for palliative care instead.

Clinical outcome assessments were conducted at the time of hospital discharge. For patients requiring extended hospitalization due to complications or delayed recovery, outcomes were evaluated at 30 days post-stroke onset.

### Statistical analysis

All data were collected from the hospital database covering the period from January 2008 to May 2023. Demographic and clinical characteristics were compared between patients with MBE and those with non-malignant brain edema (non-MBE) using the Chi-square test, Fisher’s exact test, independent t-test, or Mann-Whitney U-test, as appropriate. Radiographic factors were analyzed using univariate and multivariate logistic regression. A *p* value of less than 0.05 was considered statistically significant. To develop a clinically applicable scoring system, β-coefficients from the final multivariate logistic regression model were used to construct a weighted score [31]. All analyses were performed using IBM SPSS Statistics, version 24.0 (IBM Corp, Armonk, NY, USA).

## Results

*Patient characteristics* **(**Table 1**)**.

**Table 1:** Patient characteristics, treatments, and outcomes

Characteristics		Outcome group		*p* value
		Non-malignant brain edema (*n* = 24)	Malignant brain edema (*n* = 38)	
Demographics				
	Female, n (%)	19 (79.2)	21 (55.3)	0.055
	Age, mean ± SD	72 ± 10.5	69.3 ± 15.1	0.453
Risk factors, n (%)				
	Diabetes mellitus	8 (33.3)	12 (31.6)	0.886
	Hypertension	19 (79.2)	28 (73.7)	0.623
	Atrial fibrillation	18 (75)	24 (63.2)	0.331
	Smoking	0 (0)	5 (21.7)	0.136
Baseline clinical, mean ± SD				
	Stroke onset (Hours)	25.9 ± 2.1	25.1 ± 3.4	0.313
	SBP (mmHg)	162.7 ± 34.2	160.5 ± 24.9	0.769
	DBP (mmHg)	94.2 ± 25.2	90.5 ± 19.8	0.518
	Temperature (°C)	36.6 ± 0.7	36.6 ± 0.5	0.960
	Glucose (mg/dL)	164.0 ± 55.2	151.2 ± 49.3	0.343
	NIHSS score	19.5 ± 5.1	20.5 ± 5.6	0.485
	GCS score	11.6 ± 2.5	10.9 ± 2.7	0.311
Side of infarction, n (%)				
	Left	9 (37.5)	20 (52.6)	0.245
	Right	15 (62.5)	18 (47.4)	
Stroke type, n (%)				
	Cardioembolic	18 (75)	25 (65.8)	0.547
	Large vessel occlusion	4 (16.7)	11 (28.9)	
	Undetermined	2 (8.3)	2 (5.3)	
Treatment, n (%)				
	Intravenous thrombolysis	15 (62.5)	26 (68.4)	0.631
	Mechanical thrombectomy	4 (16.7)	4 (10.5)	0.700
	Intravenous mannitol	9 (37.5)	15 (40.5)	0.812
	Decompressive hemicraniectomy	0 (0)	16 (42.1)	< 0.001^a^
Outcome, median (range)				
	GCS score	14 (8–15)	4.5 (3–15)	< 0.001^a^
	mRS score	5 (2–5)	5 (4–6)	0.508

^a^ *p* < 0.05 indicates statistically significant difference

GCS, Glasgow Coma Scale; mRS, modified Rankin Scale; n, number; NIHSS, National Institutes of Health Stroke Scale; SD, standard deviation

In regard to the database during the study period, 345 patients with ischemic stroke were initially referred for neurosurgical consultation. Following an application of the exclusion criteria, 283 patients were excluded. They included 44 with small cerebral infarction, 14 with other coexisting intracranial mass lesions, 35 with large intracerebral hematomas, 36 with early MBE, 121 without cranial CT at 24 ± 8 h after stroke onset, 4 who underwent early prophylactic decompressive hemicraniectomy, and 29 with incomplete outcome data.

**Fig. 1:**
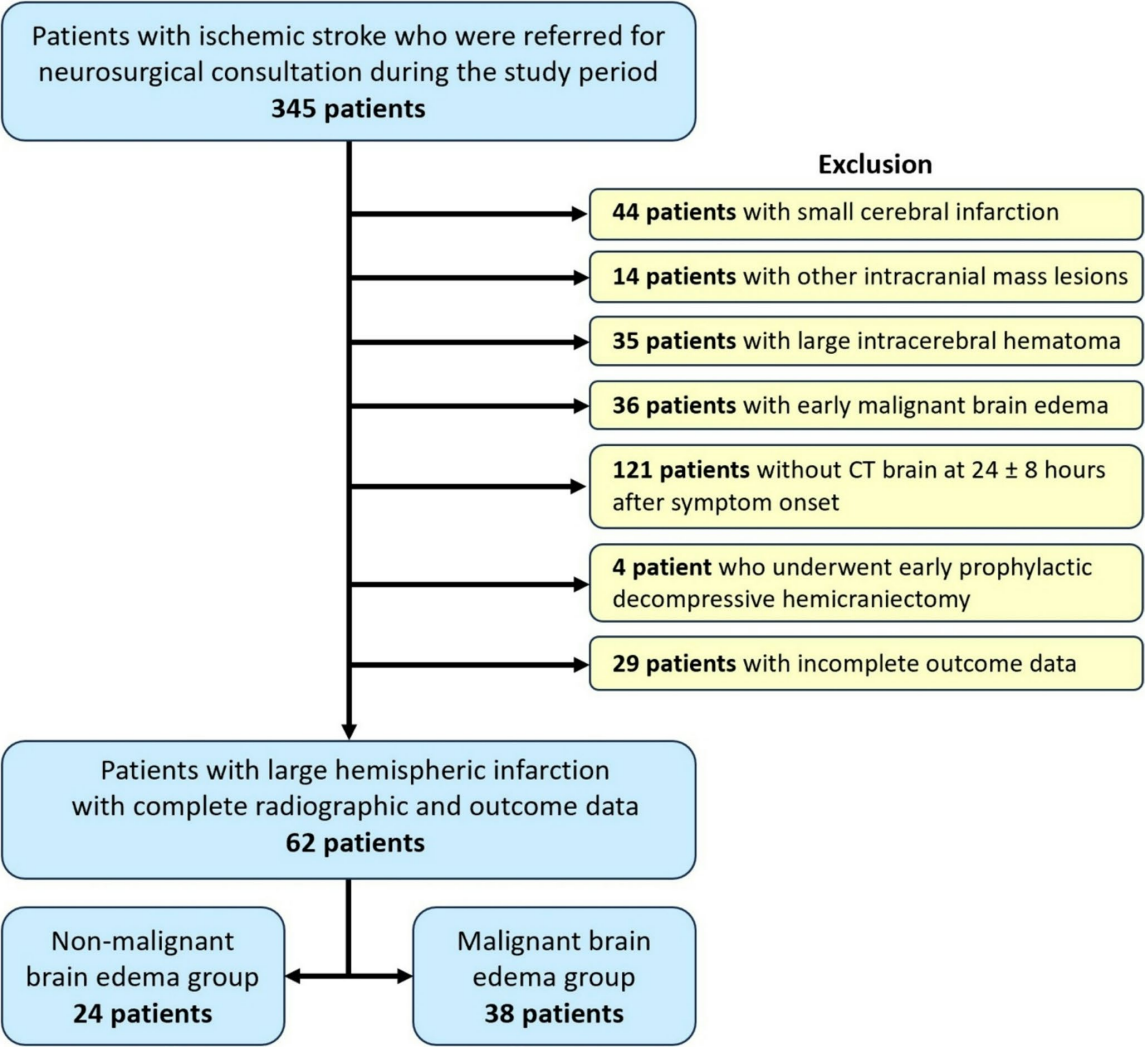
The algorithm of patient inclusion and exclusion process

Eventually, a total of 62 patients with LHI were included in our study, comprising 24 (38.7%) patients in the non-MBE group and 38 (61.3%) in the MBE group **(**Fig. 1**)**. The non-MBE group had a slightly higher mean age compared to the MBE group (72 ± 10.5 vs. 69.3 ± 15.1 years, *p* = 0.453), and females were predominant in both groups, with a higher proportion in the non-MBE group (79.2% vs. 55.3%, *p* = 0.055).

Baseline vascular risk factors such as diabetes mellitus, hypertension, and atrial fibrillation were common in both groups, with no statistically significant differences. Although a history of smoking was more frequently observed in the MBE group, the difference did not reach statistical significance (21.7% vs. 0%, *p* = 0.136). Other baseline clinical parameters, including stroke onset time, blood pressure, body temperature, serum glucose, NIHSS score, and GCS score, showed no significant differences between the two groups. There were also no differences in the side of infarction and stroke subtype between the groups.

*Treatment and outcomes* **(**Table 1**)**.

Regarding treatment, the rates of intravenous thrombolysis, mechanical thrombectomy, and IV mannitol administration were similar between groups. However, decompressive hemicraniectomy was performed exclusively in the MBE group (42.1% vs. 0%, *p* < 0.001), as expected, given its role in the management of MBE.

At discharge, patients in the MBE had significantly lower GCS scores compared to those in the non-MBE group (median 4.5 {range 3–15} vs. 14 {8–15}, *p* < 0.001). Although the mRS score was worse in the MBE group, the difference was not statistically significant (median 5 {range 4–6} in MBE group vs. 5 {2–5} in non-MBE group, *p* = 0.508).

### Radiographic factors

CT brain imaging showed meaningful radiographic differences between the MBE group and the non-MBE group **(**Table 2**)**. Infarct volume, measurement using the ABC/2 technique, was substantially larger in the MBE group (176.7 ± 63.5 ml) compared to the non-MBE group (75.3 ± 28.7 ml), with a statistical significance of *p* < 0.001. The ASPECTS was lower in the MBE group, indicating a greater ischemic area and correlating with malignant cerebral edema development (median 1 {range 0–4} in the MBE group vs. 4 {0–6} in the non-MBE group, *p* < 0.001). This finding emphasizes the significant relationship between the extent of ischemic volume and malignant events.

**Table 2:** Univariate analysis of radiographic factors

Variable		Outcome group		*p* value
		Non-malignant brain edema (*n* = 24)	Malignant brain edema (*n* = 38)	
Area of infarction				
	Infarct volume (calculated by ABC/2) (mL), mean ± SD	75.3 ± 28.7	176.7 ± 63.5	< 0.001^a^
	ASPECTS, median (range)	4 (0–6)	1 (0–4)	< 0.001^a^
Extent of infarction				
	ACA or PCA territory involvement, n (%)	4 (16.7)	19 (50)	0.008^a^
	Temporal involvement, n (%)	16 (66.7)	35 (92.1)	0.017^a^
Degree of brain edema				
	Midline shift (mm), median (range)	1.1 (0–4.1)	2.2 (0–4.8)	0.007^a^
	Lateral ventricle compression, n (%)	22 (91.7)	38 (100)	0.146
CSF space reserve				
	Intercaudate distance (mm), mean ± SD	30.2 ± 5	30.3 ± 4.3	0.899
	Sylvian effacement, n (%)	7 (29.2)	27 (71.1)	0.001^a^
	Sulcal effacement, n (%)	11 (45.8)	31 (81.6)	0.003^a^

^a^ *p* < 0.05 indicates statistically significant difference

ABC/2, estimation method for infarct volume (width × length × height ÷ 2); ACA, anterior cerebral artery; ASPECTS, Alberta Stroke Program Early CT Score; CSF, cerebrospinal fluid; n, number; PCA, posterior cerebral artery; SD, standard deviation;

Patients who suffered from malignant edema experienced infarctions in multiple vascular territories at higher rates than other patients. ACA or PCA territory involvement was observed in 50% of MBE patients, compared to 16.7% in the non-MBE group (*p* = 0.008). The MBE group displayed a significantly higher incidence of temporal lobe involvement when compared to the non-MBE group (92.1% vs. 66.7%, *p* = 0.017).

There was a notable difference in brain edema severity indicators between the groups. The median midline shift measurement was significantly greater in the MBE group at 2.2 mm compared to 1.1 mm in the non-MBE group, with statistical significance of *p* = 0.007. Both sylvian fissure effacement and sulcal effacement were also more prevalent in the MBE group, with statistical significance of 71.1% vs. 29.2%, *p* = 0.001 and 81.6% vs. 45.8%, *p* = 0.003, respectively. Lateral ventricle compression was observed in nearly every patient, but the difference between groups was not statistically significant (100% vs. 91.7%, *p* = 0.146). The intercaudate distance, a surrogate for intracranial CSF space reserve, did not significantly differ between the groups (mean 30.3 ± 4.3 mm in the MBE group compared to 30.2 ± 5 mm in the non-MBE group, *p* = 0.899).

Univariate logistic regression analysis showed significant associations between multiple radiographic factors and MBE development **(**Table 3**)**. The probability of malignant edema increased by 5% for each 1 mL increase in infarct volume (OR 1.05, 95% CI: 1.03–1.08, *p* < 0.001). Lower ASPECTS, indicating more extensive infarction, were also significantly associated with increased risk of MBE (OR 0.34, 95% CI: 0.20–0.57, *p* < 0.001). Involvement of either the ACA or PCA territories (OR 5.00, 95% CI: 1.44–17.41, *p* = 0.011) and temporal lobe infarction (OR 5.83, 95% CI: 1.37–24.94, *p* = 0.017) were identified as significant predictors of MBE. Radiographic signs of cerebral edema also showed significant associations, particularly midline shift (OR 1.80, 95% CI: 1.18–2.75, *p* = 0.007) along with Sylvian fissure effacement (OR 5.96, 95% CI: 1.94–18.37, *p* = 0.002) and sulcal effacement (OR 5.23, 95% CI: 1.66–16.49, *p* = 0.005). However, lateral ventricle compression and intercaudate distance did not show statistically significant differences between the groups. These findings highlight that infarct volume, radiographic signs of edema, and intracranial volume reserve are crucial predictors of MBE progression.

**Table 3:** Univariate logistic regression analysis of radiographic factors

Variable		Odds ratio (95% CI)	*p* value	
Area of infarction				
	Infarct volume	1.05 (1.03–1.08)	< 0.001^a^	
	ASPECTS	0.34 (0.20–0.57)	< 0.001^a^	
Extent of infarction				
	ACA or PCA territory involvement	5.00 (1.44–17.41)	0.011^a^	
	Temporal involvement	5.83 (1.37–24.94)	0.017^a^	
Degree of brain edema				
	Midline shift	1.80 (1.18–2.75)	0.007^a^	
	Lateral ventricle compression	NC	NC	
CSF space reserve				
	Intercaudate distance	1.08 (0.35–3.33)	0.897	
	Sylvian effacement	5.96 (1.94–18.37)	0.002^a^	
	Sulci effacement	5.23 (1.66–16.49)	0.005^a^	

^a^ *p* < 0.05 indicates statistically significant difference

ACA, anterior cerebral artery; ASPECTS, Alberta Stroke Program Early CT Score; CI, confidence interval; CSF, cerebrospinal fluid; n, number; NC, cannot be calculated; PCA, posterior cerebral artery

### Multivariate analysis and predictive performance

Multivariate logistic regression analysis using backward likelihood ratio elimination identified infarct volume and midline shift as significant independent predictors of MBE **(**Table 4**)**. Infarct volume remained significantly associated with malignant edema (B = 0.052, *p* = 0.003), with an adjusted odds ratio (OR) of 1.05 (1.02–1.09). This implies a 5.4% increase in MBE risk for every 1 mL increase in infarct volume. Similarly, midline shift also emerged as a significant independent predictor (B = 1.026, *p* = 0.039), with an adjusted OR of 2.79 (1.05–7.40), indicating a 2.79-fold increased risk of unfavorable outcomes for every 0.01 mm rise in midline shift. ASPECTS did not achieve statistical significance in the multivariate model, with a *p* value of 0.113.

**Table 4:** Multivariate backward logistic regression analysis of radiographic factors

Variable	Crude odds ratio (95% CI)	*p* value		Adjusted odds ratio (95% CI)	*p* value
Infarct volume	1.05 (1.03–1.08)	< 0.001^a^		1.05 (1.02–1.09)	0.003^a^
Midline shift	1.80 (1.18–2.75)	0.007^a^		2.79 (1.05–7.40)	0.039^a^
ASPECTS	0.34 (0.20–0.57)	< 0.001^a^		0.56 (0.27–1.15)	0.113

^a^ *p* < 0.05 indicates statistically significant difference

ASPECTS, Alberta Stroke Program Early CT Score; CI, confidence interval

**Fig. 2:**
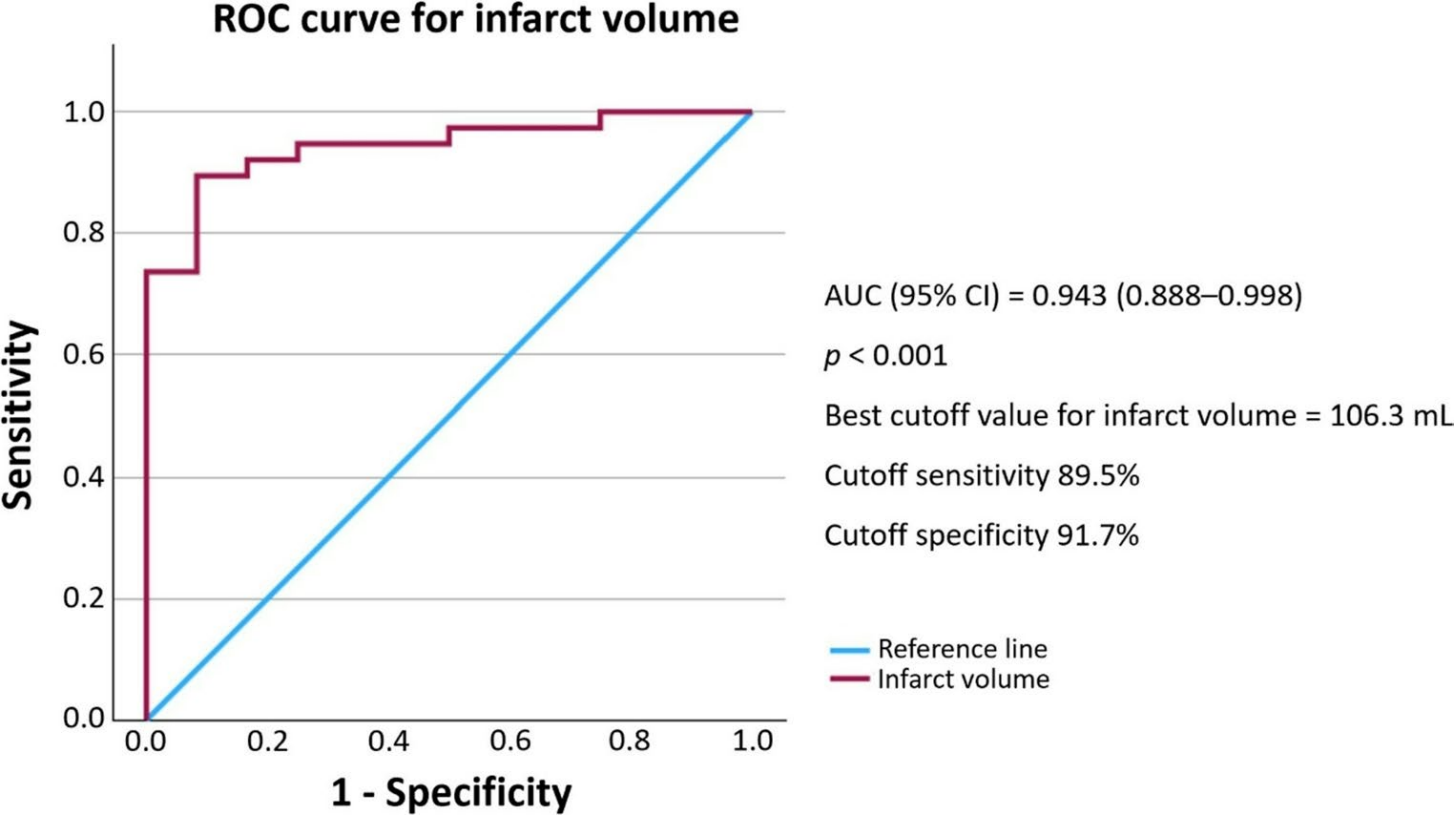
Receiver operating characteristic (ROC) curve for infarct volume AUC, area under the curve; CI, confidence interval

**Fig. 3:**
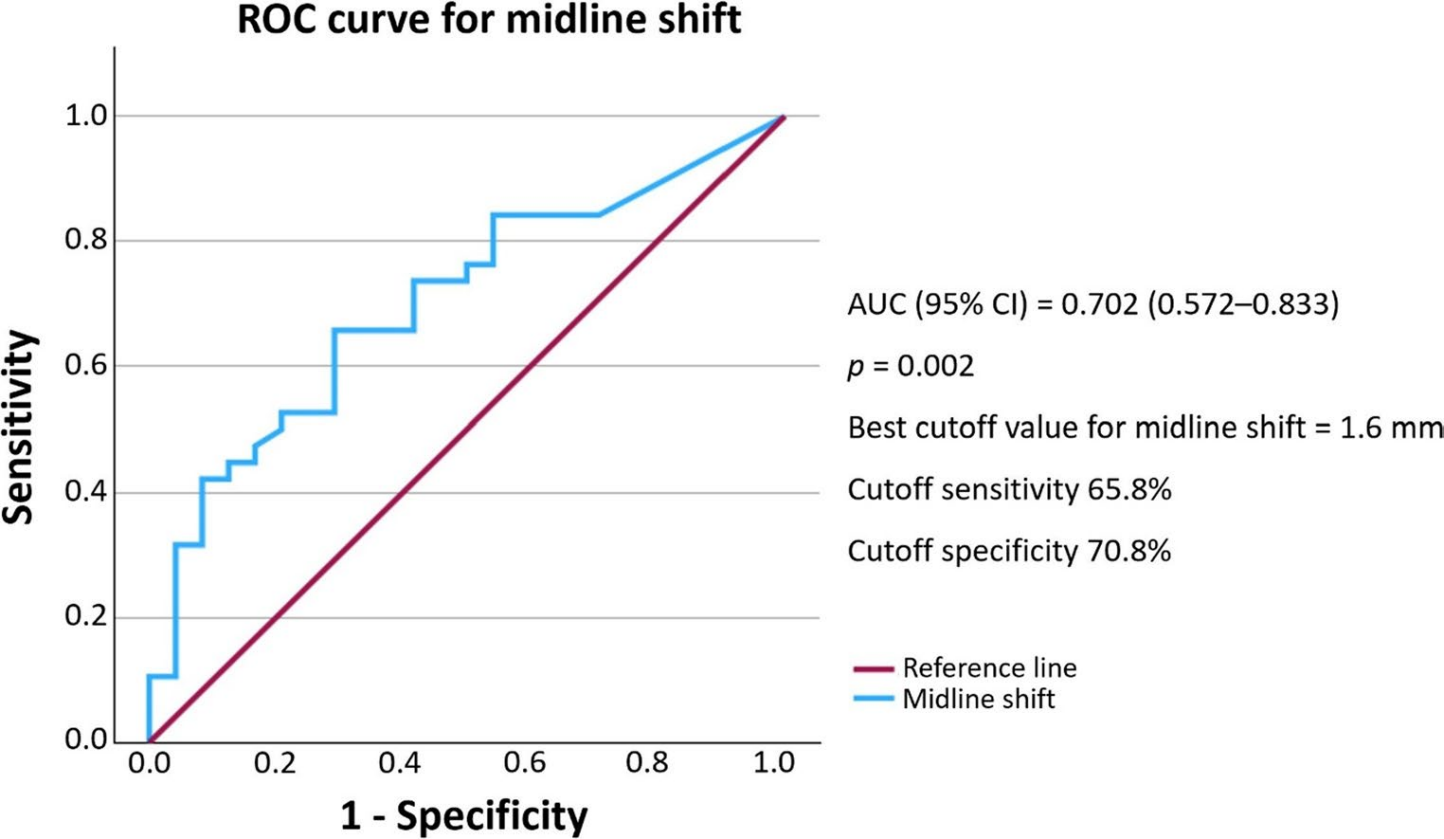
Receiver operating characteristic (ROC) curve for midline shift AUC, area under the curve; CI, confidence interval

**Fig. 4:**
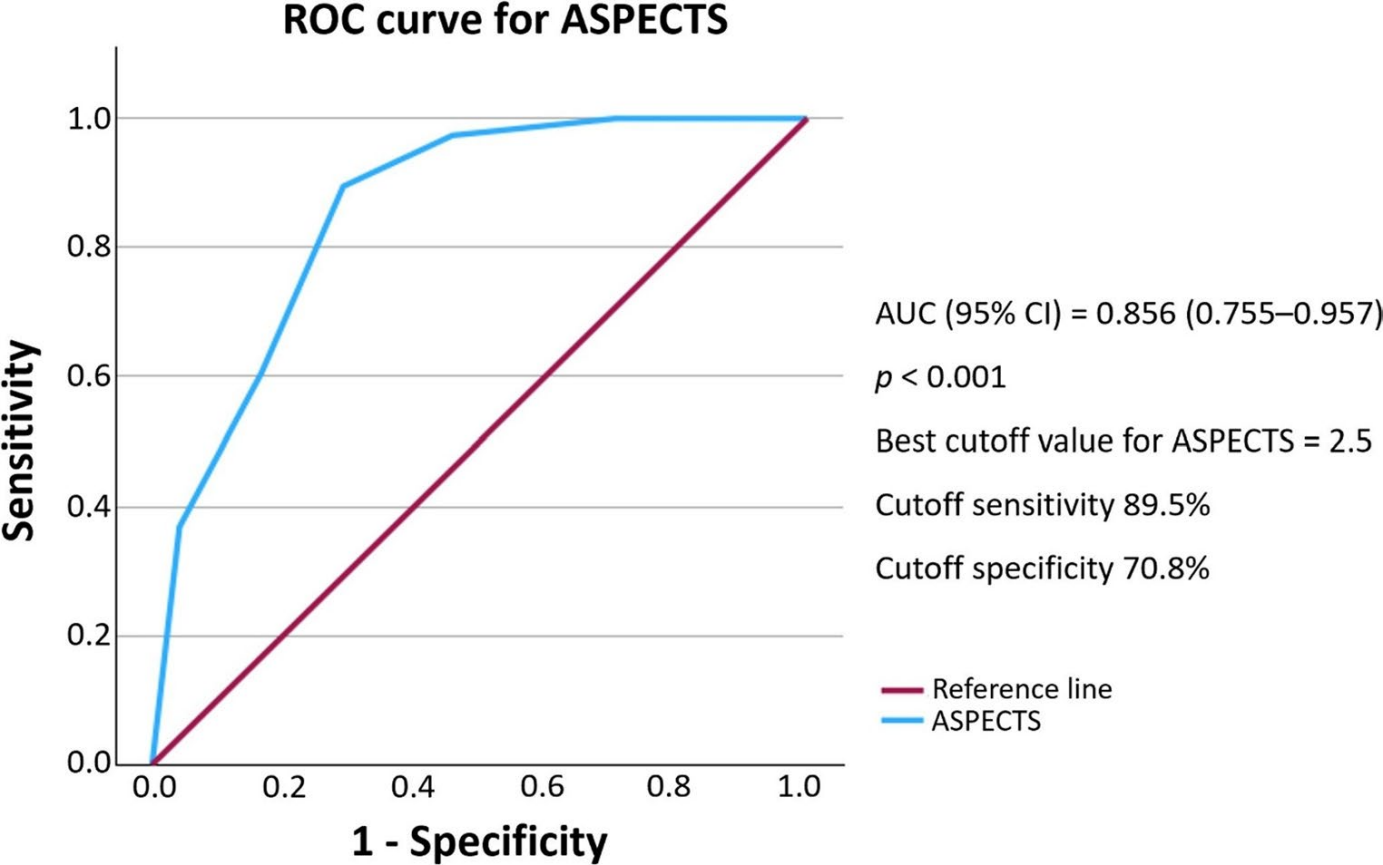
Receiver operating characteristic (ROC) curve for ASPECTS ASPECTS, Alberta Stroke Program Early CT Score; AUC, area under the curve; CI, confidence interval

The predictive performance of each radiographic variable was evaluated using receiver operating characteristic (ROC) curve analysis. Infarct volume demonstrated excellent discriminative power with an area under the curve (AUC) of 0.943 (95% CI: 0.888–0.998, *p* < 0.001). A cutoff value of 106.3 mL provided 89.5% sensitivity and 91.7% specificity for predicting malignant edema **(**Fig. 2**)**. Midline shift demonstrated moderate predictive ability, with an AUC of 0.702 (95% CI: 0.572–0.833, *p* = 0.002). A cutoff point of 1.595 mm yielded 65.8% sensitivity and 70.8% specificity **(**Fig. 3**)**. While ASPECTS was not statistically significant in the multivariate model, it exhibited good discriminatory power with an AUC of 0.856 (95% CI: 0.755–0.957, *p* < 0.001). A cutoff score of 2.5 resulted in 89.5% sensitivity and 70.8% specificity **(**Fig. 4**)**.

### Development of the scoring system

Based on the initial multivariate logistic regression analysis, infarct volume and midline shift were identified as independent radiographic predictors of MBE. To enhance clinical applicability and facilitate interpretation, these continuous variables were converted into categorical variables, with values rounded to integers for more practical use. Optimal cutoff points were determined using ROC curve analysis: infarct volume was dichotomized at 106 mL and midline shift at 1.6 mm. Additionally, ASPECTS was categorized using a cutoff of < 3. A reanalysis using backward logistic regression confirmed that infarct volume ≥ 106 mL and midline shift ≥ 1.6 mm remained as independent predictors **(**Table 5**)**.

**Table 5:** Multivariate backward logistic regression analysis of categorical radiographic factors (reanalysis)

Variable	Adjusted odds ratio (95% CI)	*p* value
Infarct volume	154.00 (15.29–1,551.52)	< 0.001^a^
Midline shift	10.51 (1.11–99.72)	0.040^a^

*p < 0.05 indicates statistically significant difference

CI, confidence interval

Weighted scores were then assigned based on the β-coefficients from the final regression model. The coefficient for infarct volume (B = 5.037) was normalized relative to the coefficient for midline shift (B = 2.353). The analysis showed that infarct volume received a relative weight of 2.14 while midline shift had a relative weight of 1.00. For simplicity, these values were rounded to whole numbers, resulting in final weights of 2 for infarct volume and 1 for midline shift. In the final scoring system, infarct volume ≥ 106 mL was assigned two points, and midline shift ≥ 1.6 mm was assigned one point. The total score ranged from 0 to three **(**Table 6**)**.

**Table 6:** The SIMPLE score: **SI**riraj **M**alignant brain edema **P**rediction by **L**esion volume and **E**dema shift

Variable	Criteria	Score
Infarct volume (mL)	< 106	0
	≥ 106	2
Midline shift (mm)	< 1.6	0
	≥ 1.6	1
Total score		0–3

The SIMPLE score is calculated by assigning 2 points for infarct volume ≥ 106 mL and 1 point for midline shift ≥ 1.6 mm, resulting in a total score range of 0–3

**Fig. 5:**
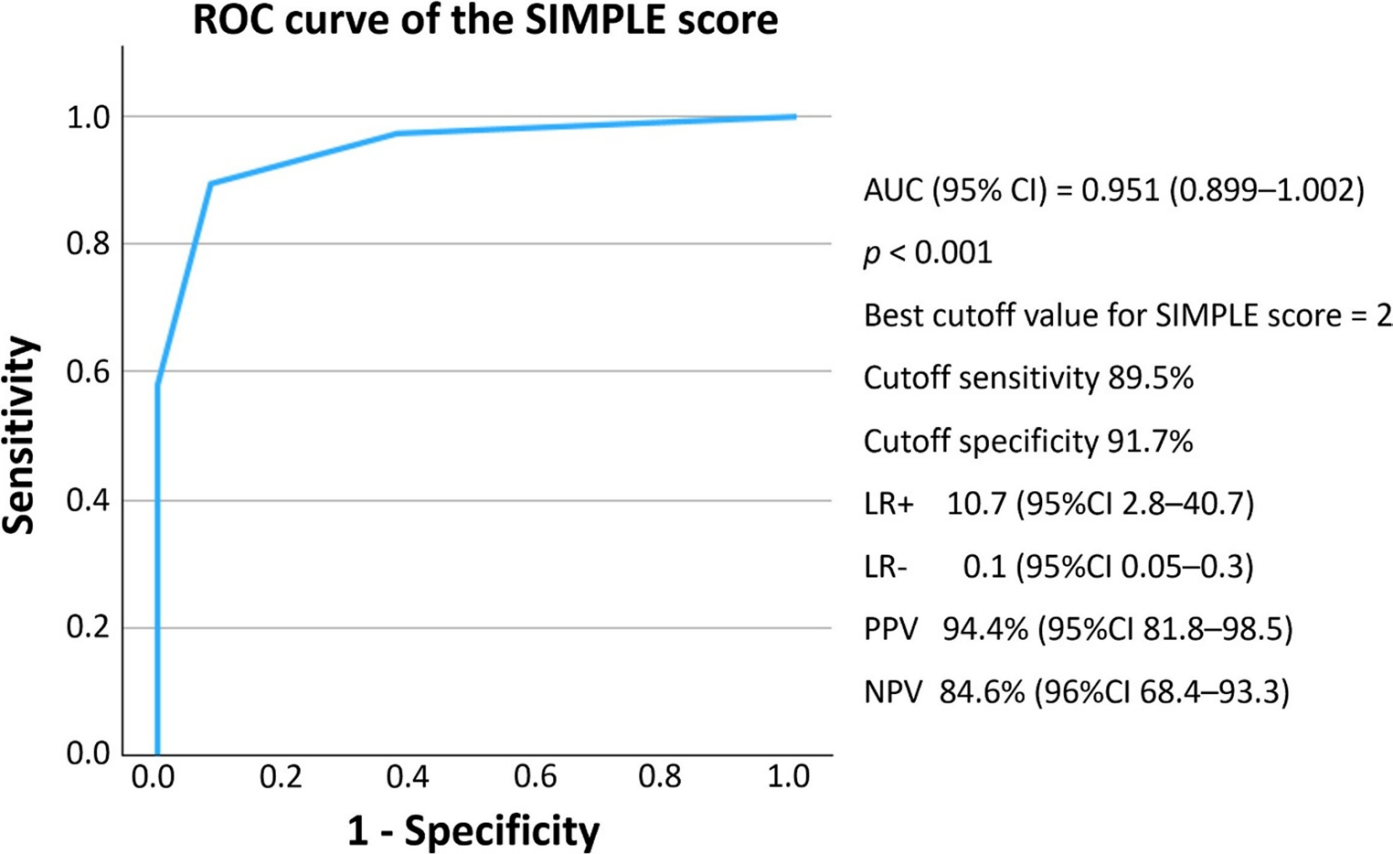
Receiver operating characteristic (ROC) curve of the SIMPLE score AUC, area under the curve; CI, confidence interval; LR-, negative likelihood ratio; LR+, positive likelihood ratio; NPV, negative predictive value; PPV, positive predictive value

The performance of this scoring system was evaluated using ROC analysis. It demonstrated excellent predictive ability, with an AUC of 0.951 (95% CI: 0.899–1.002, *p* < 0.001) **(**Fig. 5**)**. The overall model quality, as estimated by the predictive value index, was 0.90, indicating high predictive reliability. At the optimal cutoff score of two, the model achieved a sensitivity of 89.5%, specificity of 91.7%, positive likelihood ratio of 10.7 (95%CI 2.8–40.7), negative likelihood ratio of 0.1 (95%CI 0.05–0.3), positive predictive value of 94.4% (95%CI 81.8–98.5), and negative predictive value of 84.6% (96%CI 68.4–93.3). While sensitivity and specificity were comparable to infarct volume alone, the composite score showed superior discriminative performance, as evidenced by the higher AUC. This indicates that adding midline shift provides incremental value, particularly in borderline cases where subtle mass effect may be clinically relevant.

## Discussion

LHI typically follows a predictable course of progressive brain edema, with MBE developing typically within 2–5 days after symptom onset [1, 32]. Due to the dynamic and multifactorial nature of brain edema, early predictions made at admission may lack precision. We propose that non-contrast CT performed approximately 24 h after stroke onset will offer a more reliable assessment point, as it reflects both the established infarct core and the evolving degree of brain edema prior to full MBE manifestation [33]. This approach enhances predictive accuracy while utilizing a widely available imaging modality.

In this retrospective cohort of 62 patients with LHI, MBE developed in approximately 61.3% of patients. Our findings align with prior large-scale studies reporting MBE development in around 50% of LHI patients [34]. Moreover, our finding of significantly lower GCS scores at discharge in the MBE group corresponds with previous reports linking MBE to poorer neurological outcomes.

Several radiographic variables were statistically significantly associated with MBE in univariate analysis, including infarct volume, ASPECTS, ACA or PCA territory involvement, temporal lobe involvement, sulcal effacement, Sylvian fissure effacement. These results are consistent with previous studies identifying infarct size, radiographic signs of brain edema, and intracranial volume reserve as key predictive factors of MBE progression [22–27]. However, in multivariate logistic regression, only infarct volume and midline shift remained independent predictors of MBE, while other variables lost statistical significance. This finding suggests that although many radiographic signs reflect evolving cerebral edema, some may represent interrelated or secondary features rather than primary, independent risk factors.

Infarct volume emerged as the strongest predictor of MBE, in line with prior studies that established lesion size as a key factor in MBE progression [24, 26]. The influential study by Oppenheim et al. demonstrated that a diffusion-weighted imaging (DWI) lesion volume greater than 145 cm³ achieved 100% sensitivity and 94% specificity in predicting malignant middle cerebral artery infarction [35]. In our study, infarct volume of more than 106 mL, determined via ROC curve analysis, was identified as the optical cutoff for predicting MBE. This difference in volume thresholds likely represents differences in measurement methodology and imaging modality. While the ABC/2 method is practical and widely used in clinical settings, it tends to underestimate infarct volume by approximately 10% compared to true planimetric measurements [29].

Midline shift also emerged as an independent predictor of MBE. A threshold of 1.6 mm was associated with an odds ratio of 2.79 and demonstrated moderate predictive power in ROC analysis. Although this threshold is lower than the conventional definition of significant midline shift (> 5 mm), it highlights the prognostic importance of subtle early shifts before clinical deterioration or herniation becomes evident. Additionally, it serves as a simple and direct marker for MBE because it correlates with both MBE and patient outcomes [16, 27, 36, 37].

To facilitate clinical applicability, we developed the SIMPLE (**SI**riraj **M**alignant Brain Edema **P**rediction by **L**esion Volume and **E**dema Shift) score. This weighted scoring system assigns two points for infarct volume and one point for midline shift [31]. The score demonstrated excellent predictive accuracy, with a sensitivity of 89.5% and specificity of 91.7% at a cutoff score of two. Analysis of outcome distribution revealed a clear stepwise increase in MBE risk: 6.3% at score 0, 30% at score 1, 85.7% at score 2, and 100% at score 3. Notably, the only patient who developed MBE despite a SIMPLE score of 0 was a 68-year-old male (left MCA infarction) presenting with global aphasia and right hemiparesis. He initially underwent intravenous thrombolysis followed by mechanical thrombectomy. A CT scan performed at 28.3 h post-onset revealed an infarct volume of 97.4 mL and a midline shift of 1.2 mm, both just below the scoring thresholds. He later developed 6 mm midline shift but responded well to conservative treatment with intravenous mannitol and never exhibited signs of brain herniation or required surgical intervention. This borderline case highlights that even patients classified as low-risk (score 0) corresponded with a benign clinical course, reinforcing the score’s specificity and practical value in guiding non-invasive management when appropriate. However, careful clinical monitoring remains crucial in borderline presentations.

**Fig. 6:**
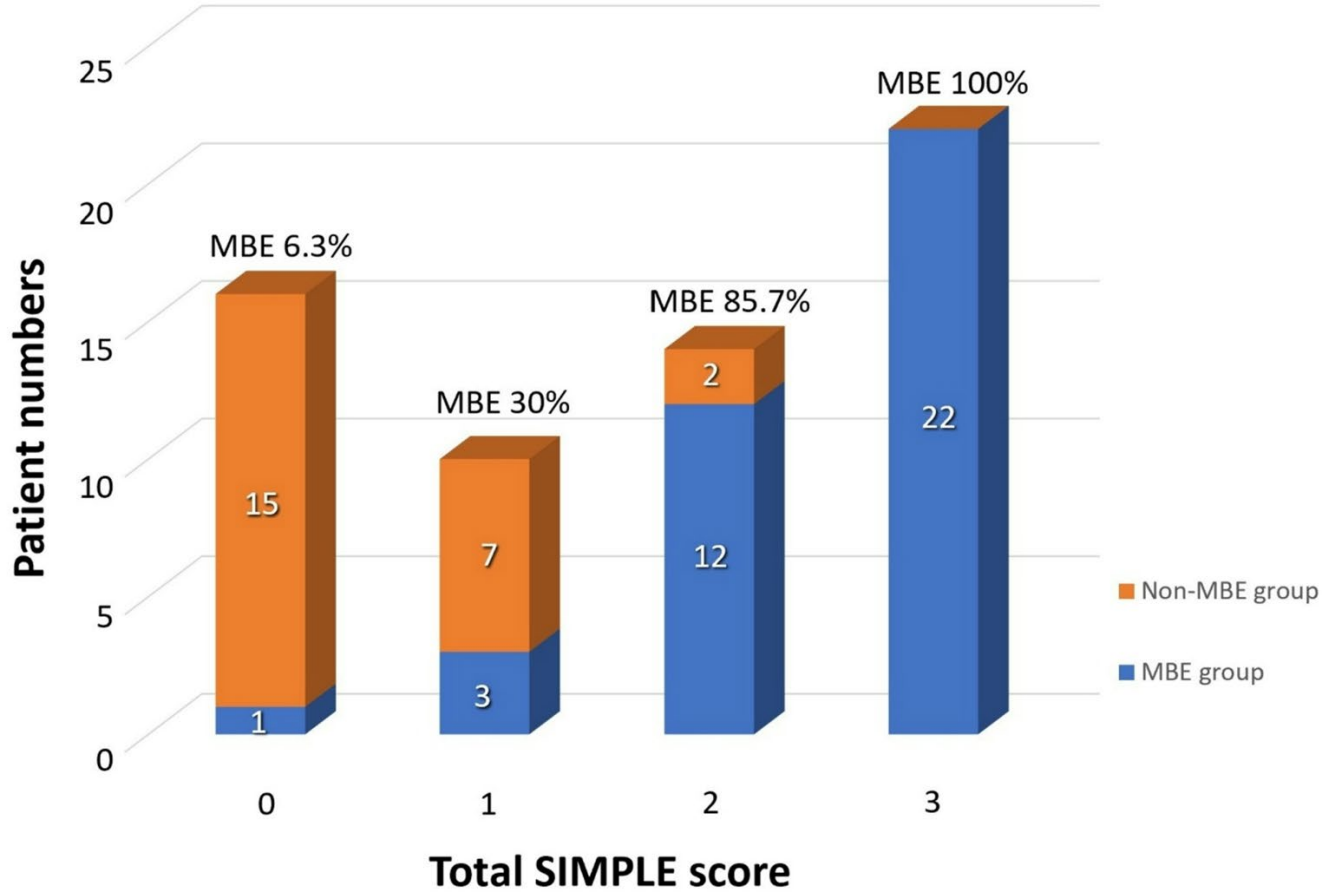
Proportion of patients with malignant brain edema (MBE) at different total SIMPLE scores. A score of three demonstrated 100% specificity for the development of malignant brain edema

**Fig. 7:**
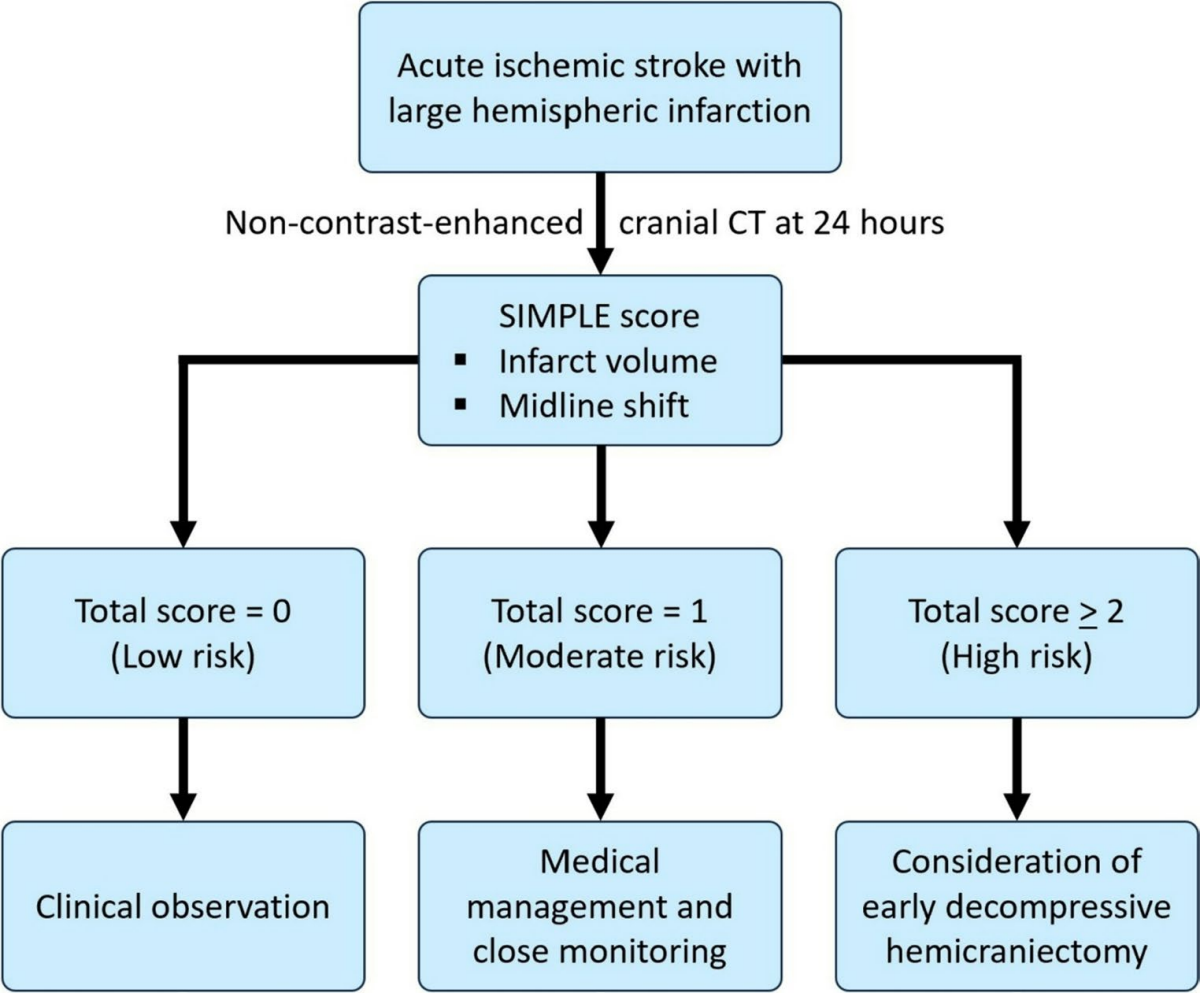
Proposed algorithm for the management of malignant brain edema based on the SIMPLE score

The SIMPLE score offers a convenient clinical tool with a dual-threshold application: a score of 0 achieves 97.4% sensitivity for ruling out MBE, while a score of three provides 100% specificity, confidently identifying high-risk cases **(**Fig. 6**)**. In comparison, other MBE risk models such as the Enhanced Detection of Edema in Malignant Anterior Circulation Stroke (EDEMA) and DASH scores adopt more comprehensive but complex approaches. The EDEMA score incorporates clinical and imaging parameters, including graded midline shift, basal cistern effacement, hyperglycemia, prior stroke history, and recanalization status, into a 14-point scale [20]. The DASH score, meanwhile, relies on advanced MRI sequences, including diffusion-ASPECTS and GRE susceptibility vessel sign, along with clinical features such as ACA involvement and blood glucose levels [16]. The SIMPLE score’s key advantages lie in its simplicity, exclusive reliance on a single routine CT scan, and high predictive reliability, making it well suited for use in a wide range of practical workflows. As illustrated in our proposed triage algorithm, the score facilitates clear decision-making pathways: patients with a score of 0 can be safely observed, those with a score of 1 may require medical management and close monitoring, and those with a score of 2–3 warrant early neurosurgical evaluation **(**Fig. 7**)**. This structure provides a reliable, evidence-based foundation for risk-guided management of LHI patients.

### Strength and limitations of study

A major strength of this study is the use of standardized imaging at a consistent time point (24 ± 8 h), a period likely to capture the final infarct alongside early edema formation, thus optimizing accuracy without sacrificing feasibility. Importantly, the model is based solely on non-contrast CT, avoiding the need for advanced imaging such as perfusion or diffusion MRI.

Regarding the limitations of this study, the initial calculated sample size was 113 cases. However, 62 patients finally met the inclusion criteria. This relatively small sample size reflects the strict imaging criteria, with a significant number of cases excluded due to lack of CT within the designated 24-hour window and exclusion of patients with early MBE. While this enhances internal consistency, it may limit statistical power and external generalizability. External validation of the SIMPLE score is warranted.

Selection bias should be mentioned as follows.

- Survivorship bias: patients who developed early MBE or died before 24 h after stroke onset were excluded. This means our study mostly contained patients who were stable enough to survive the 24-hour CT scan, and therefore the exact rate of severe outcomes may appear lower.
- Treatment-related bias: most patients who underwent 24-hour CT were those who received reperfusion therapy (intravenous thrombolysis/endovascular thrombectomy). Therefore, the analyzed population is biased toward treated or failed reperfusion cases with post-intervention CT scans, while untreated or non-intervened patients may be underrepresented.

Even though the ABC/2 method is a rapid, simple, reproducible, and accurate measure for assessment of infarct volume [29, 38, 39], it carries some limitations in use. They are described as follows.

- This approach tends to underestimate infarct volume [29, 39] or overestimate irregularly shaped infarct volume [40], and it exhibits limited precision for tiny or irregularly shaped lesions or early CT scans [29].
- The differentiation between infarct area and brain edema in a head CT is difficult. Hypodense areas can be assumed to be infarct areas, which might bring about overestimation of infarct volume [41].
- Use of the ABC/2 method in numerous, multilocular, or discontinuous lesions is limited. Instead of aggregating measurements from all the lesions, only the largest lesion was measured [38, 41].
- There is a difficulty in defining infarct margins in the acute phase, particularly less than 3 h from stroke onset, which limits use of the ABC/2 formula. Therefore, application of the ABC/2 method on non-contrast CT is more suitable in later phases of stroke, such as later than 12 h from stroke onset or last seen normal [42].

## Conclusions

This study introduces the SIMPLE score, a simple and practical radiographic evaluation tool to predict MBE in LHI patients approximately 24 h from stroke onset. Infarct volume and midline shift, both readily assessable on standard non-contrast CT, were independent predictors of MBE, and their combination in the SIMPLE score provides a balance of simplicity and accuracy. This tool offers clinicians a rapid and accessible method for early risk stratification, potentially improving patient outcomes through timely clinical decision-making. Further external validation is warranted.

## Data Availability

No datasets were generated or analysed during the current study.

## References

[1] Hacke W, Schwab S, Horn M, Spranger M, De Georgia M, von Kummer R (1996) “Malignant” middle cerebral artery territory infarction: clinical course and prognostic signs. Arch Neurol 53:309–315 8929152 10.1001/archneur.1996.00550040037012

[2] Heinsius T, Bogousslavsky J, Van Melle G (1998) Large infarcts in the middle cerebral artery territory. Etiology and outcome patterns. Neurology 50:341–350 Erratum in: Neurology 50:1940–1943 9484351 10.1212/wnl.50.2.341

[3] Vahedi K, Vicaut E, Mateo J, Kurtz A, Orabi M, Guichard JP, Boutron C, Couvreur G, Rouanet F, Touzé E, Guillon B, Carpentier A, Yelnik A, George B, Payen D, Bousser MG, DECIMAL Investigators (2007) Sequential-design, multicenter, randomized, controlled trial of early decompressive craniectomy in malignant middle cerebral artery infarction (DECIMAL Trial). Stroke 38:2506–2517 17690311 10.1161/STROKEAHA.107.485235

[4] Vahedi K, Hofmeijer J, Juettler E, Vicaut E, George B, Algra A, Amelink GJ, Schmiedeck P, Schwab S, Rothwell PM, Bousser MG, van der Worp HB, Hacke W, DECIMAL, DESTINY, and HAMLET investigators (2007) Early decompressive surgery in malignant infarction of the middle cerebral artery: a pooled analysis of three randomised controlled trials. Lancet Neurol 6:215–222 17303527 10.1016/S1474-4422(07)70036-4

[5] Jüttler E, Schwab S, Schmiedek P, Unterberg A, Hennerici M, Woitzik J, Witte S, Jenetzky E, Hacke W, DESTINY Study Group (2007) Decompressive surgery for the treatment of malignant infarction of the middle cerebral artery (DESTINY): a randomized, controlled trial. Stroke 38:2518–2525 17690310 10.1161/STROKEAHA.107.485649

[6] Hofmeijer J, Kappelle LJ, Algra A, Amelink GJ, van Gijn J, van der Worp HB, HAMLET investigators (2009) Surgical decompression for space-occupying cerebral infarction (the Hemicraniectomy After Middle Cerebral Artery infarction with Life-threatening Edema Trial [HAMLET]): a multicentre, open, randomised trial. Lancet Neurol 8:326–333 19269254 10.1016/S1474-4422(09)70047-X

[7] Wijdicks EF, Sheth KN, Carter BS, Greer DM, Kasner SE, Kimberly WT, Schwab S, Smith EE, Tamargo RJ, Wintermark M, American Heart Association Stroke Council (2014) Recommendations for the management of cerebral and cerebellar infarction with swelling: a statement for healthcare professionals from the American Heart Association/American Stroke Association. Stroke 45:1222–1238 24481970 10.1161/01.str.0000441965.15164.d6

[8] Mori K, Nakao Y, Yamamoto T, Maeda M (2004) Early external decompressive craniectomy with duroplasty improves functional recovery in patients with massive hemispheric embolic infarction: timing and indication of decompressive surgery for malignant cerebral infarction. Surg Neurol 62:420–429 15518850 10.1016/j.surneu.2003.12.017

[9] Mattos JP, Joaquim AF, Almeida JP, Albuquerque LA, Silva EG, Marenco HA, Oliveira E (2010) Decompressive craniectomy in massive cerebral infarction. Arq Neuropsiquiatr 68:339–345 20602032 10.1590/s0004-282x2010000300002

[10] Refaat MI, Abdallah OY (2018) Decompressive craniectomy in malignant middle cerebral artery infarctions: outcome of 25 cases. Egypt J Neurosurg 33:19

[11] Schwab S, Steiner T, Aschoff A, Schwarz S, Steiner HH, Jansen O, Hacke W (1998) Early hemicraniectomy in patients with complete middle cerebral artery infarction. Stroke 29:1888–1893 9731614 10.1161/01.str.29.9.1888

[12] Dasenbrock HH, Robertson FC, Vaitkevicius H, Aziz-Sultan MA, Guttieres D, Dunn IF, Du R, Gormley WB (2017) Timing of decompressive hemicraniectomy for stroke: a nationwide inpatient sample analysis. Stroke 48:704–711 28108618 10.1161/STROKEAHA.116.014727

[13] Jeon SB, Koh Y, Choi HA, Lee K (2014) Critical care for patients with massive ischemic stroke. J Stroke 16:146–160 25328873 10.5853/jos.2014.16.3.146 PMC4200590

[14] Schizodimos T, Soulountsi V, Iasonidou C, Kapravelos N (2020) An overview of management of intracranial hypertension in the intensive care unit. J Anesth 34:741–757 32440802 10.1007/s00540-020-02795-7 PMC7241587

[15] Kasner SE, Demchuk AM, Berrouschot J, Schmutzhard E, Harms L, Verro P, Chalela JA, Abbur R, McGrade H, Christou I, Krieger DW (2001) Predictors of fatal brain edema in massive hemispheric ischemic stroke. Stroke 32:2117–2123 11546905 10.1161/hs0901.095719

[16] Shimoyama T, Kimura K, Uemura J, Yamashita S, Saji N, Shibazaki K, Iguchi Y (2014) The DASH score: a simple score to assess risk for development of malignant middle cerebral artery infarction. J Neurol Sci 338:102–106 24423583 10.1016/j.jns.2013.12.024

[17] Ong CJ, Gluckstein J, Laurido-Soto O, Yan Y, Dhar R, Lee JM (2017) Enhanced detection of edema in malignant anterior circulation stroke (EDEMA) score: a risk prediction tool. Stroke 48:1969–1972 28487333 10.1161/STROKEAHA.117.016733 PMC5487281

[18] Jo K, Bajgur SS, Kim H, Choi HA, Huh PW, Lee K (2017) A simple prediction score system for malignant brain edema progression in large hemispheric infarction. PLoS One 12:e0171425 28178299 10.1371/journal.pone.0171425 PMC5298259

[19] Muscari A, Faccioli L, Lega MV, Lorusso A, Trossello MP, Puddu GM, Spinardi L, Zoli M (2019) Predicting cerebral edema in ischemic stroke patients. Neurol Sci 40:745–752 30659418 10.1007/s10072-019-3717-y

[20] Cheng Y, Wu S, Wang Y, Song Q, Yuan R, Wu Q, Zhang S, Zhang S, Wu B, Liu M (2020) External validation and modification of the EDEMA score for predicting malignant brain edema after acute ischemic stroke. Neurocrit Care 32:104–112 31549349 10.1007/s12028-019-00844-y

[21] Kimberly WT (2020) Predicting malignant cerebral edema after large hemispheric stroke. Neurocrit Care 32:84–85 31549350 10.1007/s12028-019-00841-1 PMC7018595

[22] Krieger DW, Demchuk AM, Kasner SE, Jauss M, Hantson L (1999) Early clinical and radiological predictors of fatal brain swelling in ischemic stroke. Stroke 30:287–292 9933261 10.1161/01.str.30.2.287

[23] Lam WW, Leung TW, Chu WC, Yeung DT, Wong LK, Poon WS (2005) Early computed tomography features in extensive middle cerebral artery territory infarct: prediction of survival. J Neurol Neurosurg Psychiatry 76:354–357 15716525 10.1136/jnnp.2003.035055 PMC1739552

[24] Hofmeijer J, Algra A, Kappelle LJ, van der Worp HB (2008) Predictors of life-threatening brain edema in middle cerebral artery infarction. Cerebrovasc Dis 25:176–184 18212524 10.1159/000113736

[25] Minnerup J, Wersching H, Ringelstein EB, Heindel W, Niederstadt T, Schilling M, Schäbitz WR, Kemmling A (2011) Prediction of malignant middle cerebral artery infarction using computed tomography-based intracranial volume reserve measurements. Stroke 42:3403–3409 21903965 10.1161/STROKEAHA.111.619734

[26] Wu S, Yuan R, Wang Y, Wei C, Zhang S, Yang X, Wu B, Liu M (2018) Early prediction of malignant brain edema after ischemic stroke. Stroke 49:2918–2927 30571414 10.1161/STROKEAHA.118.022001

[27] Zhang X, Huang P, Zhang R (2022) Evaluation and prediction of post-stroke cerebral edema based on neuroimaging. Front Neurol 12:763018 35087464 10.3389/fneur.2021.763018 PMC8786707

[28] Kwon SM, Choi KS, Yi HJ, Ko Y, Kim YS, Bak KH, Chun HJ, Lee YJ, Lee JY (2018) Impact of brain atrophy on 90-day functional outcome after moderate-volume basal ganglia hemorrhage. Sci Rep 8:4819 29555930 10.1038/s41598-018-22916-3 PMC5859038

[29] Sims JR, Gharai LR, Schaefer PW, Vangel M, Rosenthal ES, Lev MH, Schwamm LH (2009) ABC/2 for rapid clinical estimate of infarct, perfusion, and mismatch volumes. Neurology 72:2104–2110 19528517 10.1212/WNL.0b013e3181aa5329 PMC2697964

[30] Barber PA, Demchuk AM, Zhang J, Buchan AM (2000) Validity and reliability of a quantitative computed tomography score in predicting outcome of hyperacute stroke before thrombolytic therapy. ASPECTS Study Group. Alberta Stroke Programme Early CT Score. Lancet 355:1670–1674 10905241 10.1016/s0140-6736(00)02237-6

[31] Wilson PW, D’Agostino RB, Levy D, Belanger AM, Silbershatz H, Kannel WB (1998) Prediction of coronary heart disease using risk factor categories. Circulation 97:1837–1847 9603539 10.1161/01.cir.97.18.1837

[32] Stolz E, Gerriets T, Babacan SS, Jauss M, Kraus J, Kaps M (2002) Intracranial venous hemodynamics in patients with midline dislocation due to postischemic brain edema. Stroke 33:479–485 11823656 10.1161/hs0202.102371

[33] Park J, Goh DH, Sung JK, Hwang YH, Kang DH, Kim Y (2012) Timely assessment of infarct volume and brain atrophy in acute hemispheric infarction for early surgical decompression: strict cutoff criteria with high specificity. Acta Neurochir 154:79–85 21979162 10.1007/s00701-011-1178-z

[34] Escudero-Martínez I, Thorén M, Ringleb P, Nunes AP, Cappellari M, Rand VM, Sobolewski P, Egido J, Toni D, Chen SY, Tsao N, Ahmed N (2023) Cerebral edema in patients with severe hemispheric syndrome: incidence, risk factors, and outcomes-data from SITS-ISTR. J Stroke 25:101–110 36470246 10.5853/jos.2022.01956 PMC9911855

[35] Oppenheim C, Samson Y, Manaï R, Lalam T, Vandamme X, Crozier S, Srour A, Cornu P, Dormont D, Rancurel G, Marsault C (2000) Prediction of malignant middle cerebral artery infarction by diffusion-weighted imaging. Stroke 31:2175–2181 10978048 10.1161/01.str.31.9.2175

[36] Miao J, Song X, Sun W, Qiu X, Lan Y, Zhu Z (2020) Predictors of malignant cerebral edema in cerebral artery infarction: a meta-analysis. J Neurol Sci 409:116607 31830611 10.1016/j.jns.2019.116607

[37] Chen X, Hao Q, Yang SZ, Wang S, Zhao YL, Zhang D, Ye X, Wang H (2021) Improvement in midline shift is a positive prognostic predictor for malignant middle cerebral artery infarction patients undergoing decompressive craniectomy. Front Neurol 12:652827 34093400 10.3389/fneur.2021.652827 PMC8176305

[38] Luby M, Hong J, Merino JG, Lynch JK, Hsia AW, Magadán A, Song SS, Latour LL, Warach S (2013) Stroke mismatch volume with the use of ABC/2 is equivalent to planimetric stroke mismatch volume. AJNR Am J Neuroradiol 34:1901–1907 23449656 10.3174/ajnr.A3476 PMC4748711

[39] Sananmuang T, Dejsiripongsa T, Keandoungchun J, Apirakkan M (2019) Reliability of ABC/2 method in measuring of infarct volume in magnetic resonance diffusion-weighted image. Asian J Neurosurg 14:801–807 31497105 10.4103/ajns.AJNS_68_19 PMC6703032

[40] Pedraza S, Puig J, Blasco G, Daunis-I-Estadella J, Boada I, Bardera A, Castellanos M, Serena J (2012) Reliability of the ABC/2 method in determining acute infarct volume. J Neuroimaging 22:155–159 21447023 10.1111/j.1552-6569.2011.00588.x

[41] Arsenovic M, Rafaelian A, Dubinski D, Cantré D, Herrmann E, Behmanesh B, Trnovec S, Freiman TM, Weber MA, Wittstock M, Gessler F, Won SY (2025) Comparison of the ABC/2 formula with computer-assisted volumetry of ischemic cerebellar stroke. PLoS One 20:e0331296 40857298 10.1371/journal.pone.0331296 PMC12380265

[42] Bejleri J, Power S, Boland F, Joly O, Williams DJ, Thornton JJ, Pfeiffer S (2025) Comparative analysis of the ABC/2 score and e-ASPECTS software in the determination of acute ischaemic stroke volume from non-contrast CT. Brain Sci 15:560 40563732 10.3390/brainsci15060560 PMC12190421

